# Non-adherence to hemodialysis, perception of the illness, and severity of advanced nephropathy

**DOI:** 10.1590/2175-8239-JBN-2019-0147

**Published:** 2020-08-10

**Authors:** Lianna Gonçalves Dantas, Mario Seixas Rocha, Constança Margarida Sampaio Cruz

**Affiliations:** 1 Escola Bahiana de Medicina e Saúde Pública, Salvador, Bahia, Brasil.; 2 Clínica Senhor do Bonfim, Salvador, Bahia, Brasil.; 3 Obras Sociais Irmã Dulce, Programa de Residência de Clínica Médica, Salvador, Bahia, Brasil.

**Keywords:** Diálise, Cooperação e Adesão ao Tratamento, Falência Renal Crônica, Ajustamento emocional, Índice de Gravidade de Doença, Diálise, Cooperação e Adesão ao Tratamento, Falência Renal Crônica, Ajustamento emocional, Índice de Gravidade de Doença

## Abstract

**Introduction:**

Hemodialysis (HD) is a complex therapy that imposes several changes in the patient's life. Failure to follow therapy recommendations is called non-adherence (NA). The patient's illness perception, severity of chronic kidney disease, and individual strategies for coping with HD can have an impact on NA to the demands of therapy.

**Methods:**

This was a cross-sectional study with end-stage renal disease patients on conventional HD in Salvador, Bahia. We evaluated attendance to treatment and interdialytic weight gain (IDWG) as parameters of NA to HD, and investigated its association with clinical aspects and measures of disease perception (illness effects questionnaire - IEQ) and severity of nephropathy (end stage renal disease severity index - ESRD-SI), by analyzing Pearson or Spearman correlation.

**Results:**

79 patients were evaluated, 57% male, aged 53.1 ± 12.3 years, with length of HD of 108 (89 - 131.5) months. Age correlated with ESRD-SI (r = 0.43) and NA parameters: negative correlation with relative IDWG (r = -0.41) and reduction in sessions (r = -0.31) and positive correlation with %HD performed (r = 0.25). The scores on the IEQ and ESRD-SI showed a positive correlation (r = 0.44; p <0.001), but did not show any correlation with the analyzed NA parameters.

**Conclusions:**

We did not find a correlation between illness perception and severity index of advanced nephropathy with the behaviors of NA to chronic HD. In this study, age correlated both with the perception of severity of advanced nephropathy and the parameters of NA to chronic HD.

## Resumo

**Introdução:**

A hemodiálise (HD) é uma terapia complexa que impõe diversas mudanças à vida do paciente. O não seguimento das recomendações da terapia é denominado não aderência (NA). Percepção do paciente sobre a doença, gravidade de doença renal crônica e estratégias individuais de enfrentamento da HD podem repercutir em NA às demandas da terapia.

**Métodos:**

Estudo de corte transversal com pacientes de doença renal em estágio final em HD convencional em Salvador, Bahia. Avaliamos assiduidade ao tratamento e ganho ponderal interdialítico (GPID) como parâmetros de NA à HD, e pesquisamos sua associação com aspectos clínicos e medidas de percepção de doença (IEQ) e de severidade da nefropatia (ESRD-SI), mediante análise de Correlação de Pearson ou Spearman.

**Resultados:**

Foram avaliados 79 pacientes, 57% masculino, idade 53,1 ± 12,3 anos, em HD há 108 (89 - 131,5) meses. Idade apresentou correlação com ESRD-SI (r = 0,43) e parâmetros de NA: correlação negativa com GPID relativo (r = -0,41) e redução das sessões (r = -0,31) e correlação positiva com %HD realizada (r = 0,25). As pontuações no IEQ e ESRD-SI demonstraram correlação positiva (r = 0,44; p < 0,001), porém não apresentaram correlação com os parâmetros de NA analisados.

**Conclusões:**

Não encontramos correlação entre percepção de doença e índice de gravidade da nefropatia avançada com os comportamentos de NA à HD crônica. Neste estudo, idade teve correlação com percepção de gravidade de nefropatia avançada e parâmetros de NA à HD crônica.

## Introduction

Chronic hemodialysis (HD) treatment in end-stage renal disease (ESRD) causes important changes in the patient’s life, who is susceptible to a pathology with multiple implications in daily life and life expectancy^1^. Changes in daily habits with restrictions in food and water intake, frequent consumption of medications, in addition to dependence on a machine for preserving life are some consequences of ESRD in HD^2^^-^ 4. The demands of HD can be perceived by the patient as an interference in his/her life and lead to noncompliance with treatment recommendations and prescriptions, a fact called non-adherence (NA)^1^. There is no consensus on the most appropriate criteria for defining NA to chronic HD^5^^-^ 7.

We previously identified a stability of the behavior of NA to HD in a cohort through repeated assessments with an interval of 6 years, favoring the hypothesis that patients present behavioral patterns when faced with the demands of renal-replacement therapy (RRT)^8^. Certain psychosocial aspects of patients on RRT are associated with worse quality of life, increased morbidity and mortality, and NA to HD^7^^,^ 9 ^-^ 11.

Illness perception is a subjective issue related to quality of life, acceptance, and coping with RRT by the patient^9^^,^ 12 ^,^ 13. Disease severity is a global term that in the context of ESRD includes nephropathy and its complications, in addition to the associated comorbidities^14^. Specific questionnaires allow to objectively investigate the perception of the impact of chronic disease (Illness Effects Questionnaire -IEQ) and end-stage renal disease severity index (End Stage Renal Disease-Severity Index - ESRD-SI), which are accessible instruments for evaluation and monitoring clinical progression of HD patients^14^^,^ 15. NA to HD was associated with a worse perception of the disease and severity of nephropathy in patients in the USA^6^. In Brazil, the relationship between disease perception and severity of nephropathy has not been studied, neither has its association with NA to HD.

This study was designed to assess the association between NA to chronic HD, illness perception, severity of nephropathy, and certain clinical aspects of the patient with ESRD on dialysis.

## Methods

### Study Design and Patients

This was a cross-sectional study with chronic HD patients in Salvador, Bahia, who participated in a study of NA to HD in November 2011 and remained in the same RRT modality at the clinic 6 years later^5^. Inclusion criteria were: being on conventional HD (3 sessions/week) for at least 3 months, age ≥18 years, and absence of cognitive or psychiatric disorder. The exclusion criteria were impossibility to sign the consent form due to physical limitations and the absence of an authorized representative, and refusal to participate in the study. Of the 255 patients in the initial study, 84 remained on HD at the clinic, and 79 agreed to participate and were evaluated in November 2017 for the behaviors of NA to HD, degrees of perception of disease and severity of nephropathy, using specific questionnaires.

### Clinical and Treatment Aspects

Sociodemographic data of clinical and HD interest, etiology of CKD, and associated comorbidities (diabetes mellitus (DM), hypertension, and cardiovascular or cerebrovascular disease) were recorded. Residual diuresis was considered as the volume referred by the patient in 24 hours in the longest interdialytic period, and it was considered anuria when <100 mL/24 hours.

### Non-Adherence Parameters

The surveyed parameters of NA to HD were: interdialytic weight gain (IDWG) and attendance to treatment. The IDWG was calculated as the difference between the pre-HD weight and the weight recorded immediately after the session; the averages of sessions in one month were recorded and both absolute and relative IDWG were assessed. Relative IDWG (% IDWG) was calculated as the absolute IDWG divided by dry weight (DW) and was considered excessive if ≥4% of DW^16^. Attendance to sessions was assessed by number of missed sessions, reduced sessions, and hemodialysis length below the prescribed length per month (% HD performed)^17^. Only absences (excluding from hospitalization) and reductions in HD sessions (excluding from medical indication) per month were considered as NA.

### Instruments for Psychosocial Evaluation

#### Questionnaire on the impact of chronic disease - IEQ (Illness Effects Questionnaire)

The IEQ was adapted for Brazil in 2011, through validated translation^15^. It was developed based on the hypothesis that the patient actively thinks about the meaning of his/her illnesses, and the result of his/her assessment determines his/her behavior towards illness and therapy^15^. It consists of 20 statements to which the patient scores on a scale of 0 to 7 points according to the intensity with which he/she agrees or disagrees. The total score indicates the overall effect of the disease and ranges from 0 to 140 points, being classified as: minimum (0 to 23), mild (24 to 55), medium (56 to 88), moderate (89 to 120), or severe (120 to 140).

#### End stage renal disease severity index (ESRD-SI)

The ESRD-SI is a scoring system for recording kidney disease severity and monitoring the clinical evolution of patients undergoing chronic dialysis. It was translated and validated into Portuguese in 2004, being composed of 11 categories of pathologies and complications most commonly found in patients with CKD^14^. It encompasses the following conditions: cardiovascular, cerebrovascular, peripheral vascular, bone, respiratory and visual diseases, peripheral neuropathy, autonomic neuropathy, gastrointestinal disorders, dialysis access and events, diabetes mellitus, in addition to a residual category designated as ‘others’. Each category receives a score according to organic severity, ranging from 0 to 10 points. The total result of severity varies from 0 to 94, being classified as: absence of another disease besides CKD (0), mild (1 to 24), mild to moderate (25 to 43), moderate (44 to 58), moderate to severe (59 to 76), and severe (77 to 94).

The questionnaires were applied in a private room at the clinic, before an HD session that the patient would undergo; the patients confirmed that they were feeling good and comfortable for the interview.

### Statistical Analysis

The sociodemographic, clinical, and laboratory characteristics of the cohort were described as absolute and relative frequencies (percentages) if qualitative, and as mean ± standard deviation (SD) if continuous variables with normal distribution, or median and interquartile range (IIQ) if non-normal distribution. The prevalence of NA were expressed as percentages with respective 95% confidence intervals (95% CI). The correlations between the variables of interest were assessed with Pearson or Spearman’s correlation, according to the distribution presented. The correlation analyses were controlled for variables that could interfere with results. The level of significance adopted was p <0.05. All analyses were conducted using the SPSS version 20.0 program (SPSS Inc, Chicago, IL, USA).

## Results

Table 1 describes the sociodemographic and clinical characteristics of the 79 evaluated patients. The mean age was 53.1 ± 12.3 years, with a majority of men (57%), non-white (91.1%) and anuric (81%). The median time on HD was 108 months (89-131.5), 13.9% and 26.6% were diagnosed with DM and clinically evident cardiovascular or cerebrovascular disease, respectively.

**Table 1:** Sociodemographic and clinical characteristics of the patients.

			n = 79 (%)
Age, years			53.1 ± 12.3
		Age ≥ 65 years	19 (24.1)
Race (self-reported)		Non-white	72 (91.1)
Sex (male)			45 (57)
Civil status (married)			46 (58.2)
Treatment through the national public health service			65 (82.2)
Time on hemodialysis, months			108 (89 - 131.5)
		< 72 months	12 (15.2)
		72 - 132 months	47 (59.5)
		> 132 months	20 (25.3)
Etiology of CKD			
	Unknown		27 (34.2)
	Systemic arterial hypertension		13 (16.5)
	Glomerulonephritis		13 (16.5)
	Diabetes mellitus		9 (11.4)
	Polycystic kidney disease		10 (12.7)
	Vasculitis		2 (2.5)
	Other pathologies		5 (6.3)
HD by catheter			9 (11.4)
Active for kidney transplantation (yes)			11 (13.9)
Anuric (yes)			64 (81)
Comorbididity			
	Systemic arterial hypertension		76 (96.2)
	Diabetes mellitus		11 (13.9)
	Cardiovascular or cerebrovascular disease		21 (26.6)
Serum phosphorus (mg/dL)			4.87 ± 0.93
Serum albumin (g/dL)			3.98 ± 0.44
Kt/V			1.31 (1.15 - 1.59)
Hemoglobin (g/dL)			10.6 ± 1.02
PTHi (pg/mL)			325 (153,4 - 592)
Dry weight			67.7 ± 13.5
BMI			24.8 ± 4.47

Qualitative variables expressed in absolute values (%) and quantitative values as mean ± SD or median (1st-3rd quartile ranges) for continuous variables in accordance with distribution.

Kt/V: Fractional clearance of urea; PTHi: Parathyroid hormone; BMI: body mass index.

The prevalence of NA found and the quantitative assessment of the parameters of NA to HD are described in Table 2. The most prevalent NA results were a reduction in HD sessions and a ratio of length of dialysis performed <100%, with a frequency of 74.6 %. A minority of patients missed HD sessions (8.9%) and 32.9% had IDWG ≥4% DW. The means found for relative and absolute IDWG were 3.61 ± 1.03 and 2.41 ± 0.73, respectively.

**Table 2:** Prevalence and quantitative assessment of the parameters of non-adherence (NA) to hemodialysis (HD).

	n=79
IDWG ≥ 4% of dry weight	32.9 (22.7 - 44.4)*
% Hemodialysis performed < 100%	74.6 (63.6-83.8)*
Shortening ≥10 min of HD/month	74.6 (63.6 - 83.8)*
Skipping HD sessions/month	8.9 (3.64 - 17.4)*
Relative IDWG	3.61 ± 1.03
Absolute IDWG	2.41 ± 0.73
Shortening ≥10 min of HD/month	0.67 (0 - 1.67)
Skipping HD sessions/month	0 (0-0)
% Hemodialysis performed	99.2 (69.8 - 100)

*NA expressed as % (95%CI). Quantitative variables expressed and as mean ± SD or median (ranges from the 1st to the 3rd quartile) according to the distribution. *Value calculated with paired t-test; other variables compared to the Wilcoxon test. IDWG: interdialytic weight gain; HD: hemodialysis; %Hemodialysis performed: % relationship of hemodialysis time performed / prescribed

The score presented on the IEQ scale was 47.5 ± 24.9, with the following frequency of the overall effect of kidney disease by category: minimum (20.3%), mild (44.2%), medium (32.9 %), moderate (1.3%), and severe (1.3%). The patients had a score of 11 (5 - 20) for global disease severity by the ESRD-SI, and were classified as: no other pathology (6.3%), mild (83.6%), mild to moderate (8, 8%), moderate (1.3%), and no patient had a more severe indicative score.

**Figure 1:**
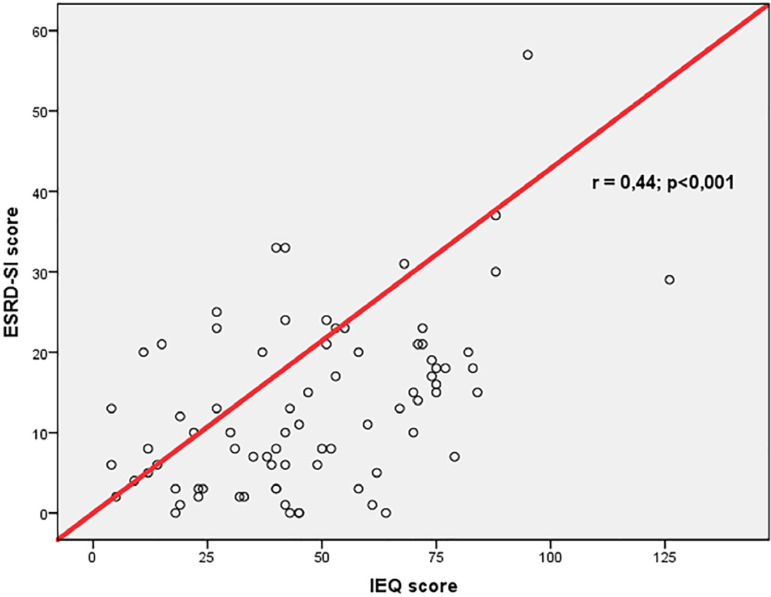
Spearman’s correlation of the ESRD-SI and IEQ scales.

Correlations between clinical and psychological aspects and NA parameters are shown in Table 3. ESRD-SI had a positive correlation with age (r = 0.43; p <0.001). IEQ and ESRD-SI showed no correlation with the NA parameters evaluated. Age had a significant correlation with NA parameters: negative correlation with %IDWG (r = -0.41; p <0.001) and reduction in sessions (r = -0.31; p = 0.005) and positive correlation with %HD performed (r = 0.25; p = 0.02). The scores on the IEQ and ESRD-SI questionnaires showed a positive correlation (r = 0.44; p <0.001). Figure 1 shows the correlation graph.

**Table 3:** Correlations between clinical and psychological aspects and non-adherence (NA) parameters.

	IEQ	ESRD-SI	Age (years)
ESRD- SI	r = 0.44	***	***
	p < 0.001		
Age (years)	r = 0.09	r = 0.43	***
	p = 0.40	p < 0.001	
Time on HD (months)	r = 0.07	r = 0.067	r = - 0.11
	p = 0.52	p = 0.55	p= 0.30
Skipping HD sessions	r = - 0.06	r = - 0.042	r = - 0.04
	p= 0.60	p= 0.71	p= 0.70
Shortening ≥10 min of HD	r = -0.02	r = -0.08	r = - 0.31
	p = 0.82	p= 0.47	p= 0.005
% IDWG	r = 0.013	r = - 0.18	r = - 0.41
	p= 0.91	p= 0.09	p < 0.001
% HD performed	r = - 0.005	r = 0.05	r = 0.25
	p= 0.96	p= 0.62	p= 0.02

Abbreviations: HD: hemodialysis; IEQ: Illness Effects Questionnaire; ESRD-SI: End Stage Renal Disease - Severity Index; % IDWG: relative interdialytic weight gain; %HD performed: HD performed/HD prescribed.

## Discussion

This study evaluated the association between certain psychological aspects and NA to chronic HD therapy after a long period of dialysis, in a sample of patients who had stability in NA behaviors after 6 years in therapy^8^. We found no correlation between the behaviors of NA to HD surveyed and the patient’s perception of the disease and severity of advanced nephropathy.

Poor adherence to HD is a complex issue that can be evidenced as a reduction in attendance at sessions with a consequent reduction in the dialysis dose^17^^,^ 18. Some hypotheses are suggested to explain the motivations for reduced attendance to HD. The patient’s perception that he/she exercises control over the chronic treatment can motivate the occurrences of absences and reductions in the HD sessions^1^^,^ 5 ^,^ 19. Another possibility is that the rigidity of times for performing HD combined with the time spent in sessions and the occurrence of symptoms after HD, including slow recovery after treatment, are considered motivators for absences, despite the potential risks to patients^6^^,^ 8 ^,^ 20.

In light of the fact that the HD patient receives individualized supervision, guided by residual renal function (RRF) and nutritional condition, excessive IDWG means failure to comply with these recommendations and risk of hypervolemia with potential complications^8^^,^ 21 ^,^ 22. About a third of the patients had IDWG ≥ 4% DW and 81% had anuria, which can be justified by the long period on HD (108 months). Anuria contributes to higher IDWG and is a factor related to higher mortality in HD since RRF contributes to clearance of medium molecules and solutes linked to plasma proteins^23^^,^ 24. In the cohort study that assessed NA to HD and survival, patients had similar IDWG and % IDWG after 6 years on HD (%IDWG of 3.48 ± 1.34 and 3.61 ± 1.03 in 2011 and 2017, respectively), despite the difference found in residual diuresis (p <0.001), demonstrating a reduction in intake when facing loss of RRF^8^.

The diagnosis of chronic nephropathy triggers in a patient a sequence of events that involve understanding, elaboration, and coping with the disease and its complications, called perception of the illness^12^^,^ 13. Through the IEQ, we identified a perception predominantly in the minimum to light levels (63.3%), and only 2.6% of the patients obtained moderate to severe scores. The mean IEQ found was lower than that of other studies and may result from the long period on HD, therefore representing a group of patients who have already accepted the disease and which consequently demonstrate a lower perception of treatment interference in their lives^3^^,^ 25.

We found a positive correlation between IEQ and ESRD-SI that can be explained by the worse perception of the illness in individuals with the most compromised global condition^5^. In a cohort of 118 patients, Fonseca assessed the correlation between IEQ, ESRD, and BDI (Beck’s depression inventory) and identified a positive correlation between ESRD-SI and IEQ scores (r = 0.26) and between BDI and IEQ (r = 0.68)^26^. We found overall severity of the disease to be predominantly mild (82.3%) and no patient showed moderate-severe or severe scores, and this fact can be justified by the presence of patients with fewer comorbidities. The low prevalence of DM and cardiovascular and cerebrovascular diseases in this sample corroborates this hypothesis. We identified a positive correlation between age and disease severity (r = 0.44) despite the ESRD-SI result with a low severity score. Age correlated with NA parameters, and previous studies have shown that young patients have worse adherence to HD^5^. The young person on dialysis may feel less vulnerable to the complications of the disease and chronic therapy and, in addition, perceive HD as an intervention in the routine and purpose of life^1^^,^ 4.

The IEQ and ESRD-SI questionnaires were translated and validated to a Brazilian version with high levels of reliability for adult patients on chronic HD^14^^,^ 15. The IEQ is a self-applied instrument, but due to the limited visual acuity and low academic level of our sample, it was read for all patients. The questionnaires were applied by the same professional and in an environment of total privacy so as not to compromise the results.

Our sample consisted of a majority of non-white patients, on dialysis for arteriovenous fistula, and without supplementary health insurance, data that reflect the reality of many HD services in Brazil^27^. However, the prevalence of DM found (13.9%) was lower than that registered in Brazil (31%), as well as the proportion of elderly people (24.1% versus 34.3%) according to data from the Brazilian Dialysis Registry^27^. Our cohort was composed of patients on HD for more than 6 years, which explains the lower prevalence of the elderly and diabetics, who are subgroups of patients known to have lower survival in chronic HD^28^.

The present study has several limitations. First, our sample consisted of patients with long-term HD, adequate vascular access and metabolic control and, therefore, does not represent the context of incident patients on dialysis or with a very compromised global health condition. Second, the IEQ and ESRD-SI assessments were carried out in a single moment and, therefore, do not reflect seasonal variations and the evolution of these results over the years in HD. Third, the low IEQ and ESRD scores obtained compromise the generalization of data about the effect of these psychological variables on the patient’s behavior in chronic HD. However, these findings are indicative that, for patients on HD for many years, NA to therapeutic demands has no association with the patient’s perception of their illness and severity of nephropathy, but may be associated with other variables. Understanding the psychosocial aspects and other variables related to NA to HD can guide behaviors for better acceptance and follow-up of RRT.

## Conclusion

We did not find a correlation between the patient’s perception of his/her illness and the severity index of advanced nephropathy with non-adherence behaviors to chronic hemodialysis. In this study, age correlated with the perception of severity of advanced nephropathy and parameters of non-adherence to hemodialysis.
